# Sense of Coherence as a Moderator Between Social Isolation and the Risk of Care Dependency Among Older Adults in Japan

**DOI:** 10.3390/nursrep15110403

**Published:** 2025-11-17

**Authors:** Shimpei Hayashi, Keiko Matsumoto

**Affiliations:** Department of Home Care Nursing, Faculty of Medicine, Kagawa University, 1750-1 Ikenobe, Miki-cho, Kita-gun, Kagawa 761-0793, Japan; matsumoto.keiko@kagawa-u.ac.jp

**Keywords:** social isolation, sense of coherence, care dependency, older adults, community setting

## Abstract

**Background/Objectives:** In Japan, the rapid aging of the population has increased the need for strategies to extend healthy life expectancy and prevent care dependency. Social isolation has been identified as a major risk factor for adverse physical and psychological outcomes, but its interaction with psychological resilience factors remains unclear. This study aimed to examine the association between social isolation and the risk of care dependency among community-dwelling older adults, and to investigate whether the sense of coherence (SOC) moderates this relationship. **Methods:** A cross-sectional survey was conducted in City A, Kagawa Prefecture, involving 519 residents aged 65 years or older. Social isolation was assessed using the Japanese version of the Lubben Social Network Scale-6 (LSNS-6), and SOC was measured with a validated three-item scale from the University of Tokyo. The risk of care dependency was evaluated using a 15-item checklist developed by the Tokyo Metropolitan Institute of Gerontology. Nutritional status was measured using the Mini Nutritional Assessment–Short Form. Multiple imputation (*m* = 50) handled missing data. Standardized linear regression analyses estimated main and interaction effects, followed by robustness checks using robust, gamma, and bootstrap analyses. **Results:** Lower levels of social connectedness were associated with a higher risk of care dependency. A moderating trend of SOC was observed (β = 0.100, *p* = 0.004), suggesting that the adverse impact of social isolation may be greater among individuals with lower SOC. **Conclusions:** These findings suggest that SOC may play a potential buffering role mitigating the adverse effects of social isolation. Although the explanatory power of the model was moderate, the observed trends highlight the potential importance of psychosocial resources for preventive care among older adults.

## 1. Introduction

Population aging is accelerating globally, and Japan represents one of the most rapidly aging societies in the world. Extending healthy life expectancy and preventing care dependency have become urgent public health priorities. Identifying the factors that contribute to care needs before older adults require long-term care is essential for timely intervention and the establishment of a sustainable care system. These risk factors are multifactorial, reflecting the complex interplay among physical, psychological, and social components that intensify with age [1].

Among these factors, social isolation has gained increasing attention as a critical determinant of health. Previous studies have linked social isolation to a wide range of adverse outcomes, including increased mortality, depressive symptoms, functional decline, and limitations in activities of daily living [2,3,4]. In Japan, the proportion of older adults living alone continues to rise. According to the White Paper on the Aging Society (2024), 15.0% of men and 22.1% of women aged 65 years and older were living alone in 2020, and these rates are projected to increase to 26.1% and 29.3%, respectively, by 2050 [5]. Against this backdrop, preventing social isolation has become a national concern, especially amid growing shortages of care workers that threaten the sustainability of the long-term care system.

To address these challenges, Japan’s national policies explicitly emphasize the principles of self-help and mutual support, highlighting the importance of strengthening older adults’ self-management abilities and internal psychological resources [6]. Similarly, the World Health Organization (WHO, 2015) identified intrinsic capacity, resilience to stress, and self-directed behavior as key elements of healthy aging, underscoring the theoretical and practical importance of mobilizing internal resources in addition to external support systems [7].

One such internal resource is the Sense of Coherence (SOC), a concept proposed by Antonovsky [8]. SOC represents a psychological orientation that enables individuals to perceive life as comprehensible, manageable, and meaningful. A growing body of evidence demonstrates that older adults with higher SOC tend to exhibit better physical and mental health, including lower risks of chronic illness, depression, and functional impairment [9,10,11,12,13,14,15]. These findings suggest that SOC functions as a protective psychological factor against age-related health decline.

Recent studies have explored whether SOC also mitigates the negative consequences of social isolation. Several investigations have reported a protective role of SOC. For example, Giglio et al. [10] found that higher SOC was associated with better health-related quality of life among community-dwelling older adults in Spain, while Adebusoye et al. [15] reported similar findings in Nigerian populations. These results imply that SOC may serve as a health-promoting psychological resource, particularly among socially vulnerable groups.

However, the buffering role of SOC has not been consistently demonstrated. Van Woerden et al. [16] reported that SOC buffered the adverse effects of long-term conditions and disability on quality of life, but not those of loneliness or social isolation. Similarly, Lin et al. [17] found that SOC mediated—but did not moderate—the relationship between aging-related self-stereotypes and social isolation. Park et al. [18] further observed that among older Korean Americans, the harmful effects of social isolation were mitigated more by family and community cohesion than by SOC alone. These inconsistencies suggest that the protective role of SOC may vary across individual, cultural, and environmental contexts, warranting further investigation.

Based on these theoretical perspectives and prior research, this study aimed to examine the association between social isolation and the risk of care dependency among community-dwelling older adults in Japan and to investigate whether SOC moderates this relationship. We hypothesized that SOC would buffer the negative association between social isolation and care dependency, such that the association would be weaker among individuals with higher SOC.

## 2. Materials and Methods

### 2.1. Study Design and Participants

This cross-sectional study targeted community-dwelling older adults aged ≥65 years residing in a district of City A, Kagawa Prefecture, Japan. The survey area corresponded to a neighborhood federation that combined both urban and rural characteristics. Participants were identified from the federation roster. As of April 2023, 726 eligible residents aged ≥65 years were invited to participate in a complete census survey targeting all older adults registered in the neighborhood federation. Because the study aimed to include the entire population of older residents within the federation, no a priori sample size calculation was performed. Although a full enumeration was attempted, the obtained dataset was treated as a sample due to non-response and other exclusions.

Eligibility included all older adults living in the community, regardless of household composition. Individuals with severe cognitive impairment or psychiatric conditions preventing independent questionnaire completion were excluded. Written information describing the study’s purpose, procedures, voluntary participation, and data protection was provided in advance, and written informed consent was obtained from all participants. The study was approved by the Ethics Committee of the Faculty of Medicine, Kagawa University (Approval No. 2023-027).

### 2.2. Measures

#### 2.2.1. Social Isolation

Social isolation was assessed using the Japanese version of the Lubben Social Network Scale-6 (LSNS-6) [19], a validated measure of perceived social connectedness among older adults. The original abbreviated version of the LSNS-6 was developed by Lubben et al. [20]. The LSNS-6 comprises six items—three assessing family ties and three assessing friendships—each rated on a 6-point scale (0–5). Total scores range from 0 to 30, with lower scores indicating stronger social isolation. In this study, lower LSNS-6 scores were operationalized as indicators of social isolation, consistent with prior research defining isolation as limited interpersonal interaction and network size [21]. Although formal support is not included, the LSNS-6 effectively captures informal social connectedness, aligning with international efforts to standardize measurement of isolation [22].

#### 2.2.2. Sense of Coherence (SOC)

SOC was measured using the Japanese version of the SOC-3-UTHS, developed at the University of Tokyo [23]. This brief scale includes three items representing comprehensibility, manageability, and meaningfulness. Each is rated on a 7-point scale (total range = 3–21); higher scores indicate a stronger SOC. The short form was selected for its reliability, validity, and suitability for older populations, while minimizing respondent burden given the comprehensive nature of the questionnaire.

#### 2.2.3. Nutritional Status

Nutritional status was evaluated using the Japanese version of the Mini Nutritional Assessment-Short Form (MNA-SF) [24,25]. The MNA-SF consists of six items covering food intake, weight loss, mobility, psychological stress or acute illness, neuropsychological problems, and either body-mass index or calf circumference. Scores range 0–14, with ≥12 = normal nutritional status, 8–11 = risk of malnutrition, ≤7 = malnourished. Poor nutritional status has been linked to frailty, functional decline, and higher risk of care dependency [26,27]; therefore, MNA-SF scores were included as an independent variable reflecting physiological vulnerability.

#### 2.2.4. Risk of Care Dependency

The 15-item Care Prevention Checklist, developed by Shinkai et al. [28], was used to assess risk of care dependency. Although initially designed to screen for frailty, this tool is widely accepted as a multidimensional proxy for future care needs. It evaluates physical, nutritional, cognitive, psychological, sensory, lifestyle, and social participation domains. Each item is binary, with total scores 0–15; higher scores indicate greater risk. The validity of this checklist has been demonstrated across multiple studies, including longitudinal research showing associations with subsequent disability and mortality [27,29]. Other indices (e.g., Barthel Index [30] and Lawton IADL scale [31]) were reviewed but focus primarily on physical function. The 25-item Basic Checklist of Japan’s Ministry of Health, Labour and Welfare was also considered but deemed overly lengthy and less sensitive to psychosocial aspects. The 15-item version was therefore chosen for its balance of comprehensiveness and feasibility. Data for the Barthel Index and Lawton’s IADL scale were not collected, as these tools were reviewed only for comparison and were not included in the present analysis.

### 2.3. Data Collection

Data were collected between October and November 2023 in cooperation with neighborhood associations. Paper-based, self-administered questionnaires were distributed by trained local volunteers and returned via mail, direct hand-back, or submission to the neighborhood office. Of 726 distributed questionnaires, 519 were returned, yielding a valid response rate of 71.5%. Each questionnaire was accompanied by an information sheet explaining study aims, voluntary participation, and confidentiality. Written informed consent was implied upon submission of the completed form.

### 2.4. Missing Data Handling

Missing data for key variables (e.g., LSNS-6, MNA-SF, age) were handled using multiple imputation by chained equations (MICE) in R. Fifty imputations (*m* = 50, seed = 1111) were conducted for the main analysis and twenty (*m* = 20, seed = 2025) for sensitivity testing. All analytical variables (excluding interaction terms) were included in the imputation model. Predictive mean matching was used for continuous variables, and logistic regression for categorical variables. Estimates from imputed datasets were pooled using Rubin’s rules. Comparisons of pre- and post-imputation means and standard deviations confirmed negligible change, and visual inspection revealed no distortion of distributions; bias from imputation was therefore considered minimal.

### 2.5. Statistical Analysis

Analyses were performed using R version 4.3.3 (R Foundation for Statistical Computing, Vienna, Austria) [32]. Multiple imputation was conducted using the mice package (version 3.16.0; https://cran.r-project.org/package=mice (accessed on 12 November 2025)).

Robust regression was performed using the MASS package (version 7.3-60; https://cran.r-project.org/web/packages/MASS/index.html (accessed on 12 November 2025)), and generalized linear models were implemented using the stats package in R.

Continuous variables (LSNS-6, SOC, MNA-SF, age, Care Prevention Checklist score) were standardized (Z-scores).

Descriptive statistics for all variables are shown in Table 1. A basic linear regression model examined associations between LSNS-6, SOC, MNA-SF, age, sex, and living arrangement (living alone vs. not) and the Care Prevention Checklist score. To test moderation, an extended model added the interaction term (LSNS-6 × SOC). Model fit was compared using the Akaike Information Criterion (AIC) and adjusted R^2^. The robustness of the findings was evaluated using multiple complementary approaches, including sensitivity analyses with varying numbers of imputations, robust regression with Huber’s M-estimator to account for outliers, and a generalized linear model with a gamma distribution and a log link, because the dependent variable was strictly positive and right-skewed. Bootstrap validation based on 1000 resamples was also performed. Multiple imputation using chained equations was performed to handle missing data, generating 50 imputed datasets (*m* = 50). Slight differences in the estimates between models using *m* = 50 (Table 2, Model 2; Table 3 and Table 4) are attributed to statistical variations resulting from the use of different random seed values during the imputation process. Specifically, the datasets for Table 2 and Table 4 were generated with seed = 1111 (main analysis), whereas Table 3 used seed = 2025 as an independent robustness check. These independent imputation runs were performed intentionally to confirm that the results were not dependent on a specific seed value. The direction and significance of the key findings remain consistent, confirming the robustness of the analysis. Interaction effects were further examined and visualized by plotting the predicted values using standardized datasets.

## 3. Results

### 3.1. Participant Characteristics

Table 1 summarizes the characteristics of the 519 participants.

**Table 1:** Characteristics of the Participants (N = 519).

Variable	Value (Mean ± SD or n [%])
Age	77.89 ± 7.61
Female	354 (68.2)
Living alone	155 (29.9)
Social network (LSNS-6)	14.91 ± 6.03
Sense of coherence (SOC)	15.65 ± 3.61
Nutritional status (MNA-SF)	11.68 ± 2.48
Risk of Care dependency (Care Prevention Checklist)	3.14 ± 2.21

Note. LSNS-6 = Lubben Social Network Scale–6 [19,20]; SOC = Sense of Coherence, measured using the Japanese version of the SOC-3-UTHS, developed by the University of Tokyo [23]; MNA-SF = Mini Nutritional Assessment–Short Form [24,25]; Care Prevention Checklist = 15-item screening tool [28].

The mean age was approximately 78 years, and about 30% lived alone. Although the proportion of individuals living alone differs slightly from national estimates, this study focused on associations among psychosocial and health-related variables rather than population prevalence. Descriptive statistics (means ± standard deviations) for the main study variables—LSNS-6, SOC, MNA-SF, and Care Prevention Checklist scores—are presented in Table 1.

### 3.2. Main Analysis

Table 2 presents the results of the linear regression models conducted with and without the interaction term (*m* = 50; estimates pooled using Rubin’s rules).

**Table 2:** Comparison of Linear Regression Models: With and Without Interaction Term (*m* = 50).

		Model 1			Model 2	
Variable	β	SE	*p*-Value	β	SE	*p*-Value
Intercept	0.027	0.068	0.696	−0.002	0.068	0.976
LSNS-6	−0.251	0.041	<0.001 ***	−0.248	0.041	<0.001 ***
SOC	−0.119	0.04	0.003 **	−0.116	0.04	0.004 **
MNA-SF	−0.221	0.046	<0.001 ***	−0.224	0.046	<0.001 ***
Age	0.263	0.043	<0.001 ***	0.25	0.043	<0.001 ***
Female (ref: male)	−0.03	0.085	0.724	−0.032	0.084	0.709
Living alone	0.036	0.095	0.706	0.041	0.095	0.665
LSNS-6 × SOC	—	—	—	0.1	0.035	0.004 **

Note. All continuous variables (LSNS-6, SOC, MNA-SF, Age) were standardized (Z-transformed) before analysis. LSNS-6 = Lubben Social Network Scale–6; SOC = Sense of Coherence; MNA-SF = Mini Nutritional Assessment–Short Form; SE = Standard error. “—” indicates that the variable was not included in the model. ** *p* < 0.01; *** *p* < 0.001. Adjusted R^2^ = 0.291 (Model 1), 0.302 (Model 2); AIC = 1156.13 (Model 1), 1149.63 (Model 2). AIC = Akaike Information Criterion. Lower values indicate better model fit. The small AIC difference supports model integration.

In the basic model, LSNS-6, SOC, MNA-SF, and age were significant predictors of care dependency risk, whereas sex and living arrangement were not. In the extended model, the interaction between LSNS-6 and SOC reached statistical significance, indicating that the protective association of social connectedness with lower care dependency risk was stronger among participants with lower SOC.

### 3.3. Model Comparison: With vs. Without the Interaction Term

Model fit was compared using the Akaike Information Criterion (AIC) and adjusted R^2^ values. As shown in Table 2, both indices slightly favored the extended model, supporting the inclusion of the interaction term. Subsequent sensitivity and robustness analyses were therefore based on this extended model structure.

### 3.4. Robustness Analyses

Several supplementary analyses were performed to confirm the robustness of the findings. First, sensitivity analyses assessed whether varying the number of imputations affected the results. Re-estimation of the interaction model using a reduced number of imputations (*m* = 20, seed = 2025) produced coefficients and significance levels comparable with the main analysis (*m* = 50), confirming stability across imputation settings (Table 3).

**Table 3:** Comparison of Regression Coefficients Between Main and Sensitivity Analyses.

Variable	β (*m* = 50)	SE	*p*-Value	β (*m* = 20)	SE	*p*-Value
Intercept	−0.034	0.069	0.624	−0.033	0.069	0.638
LSNS-6	−0.281	0.041	<0.001 ***	−0.285	0.04	<0.001 ***
SOC	−0.103	0.041	0.013 *	−0.1	0.041	0.014 *
MNA-SF	−0.158	0.041	<0.001 ***	−0.159	0.041	<0.001 ***
Age	0.239	0.043	<0.001 ***	0.239	0.043	<0.001 ***
Female (ref: male)	−0.009	0.085	0.92	−0.009	0.085	0.918
Living alone	0.047	0.094	0.617	0.045	0.094	0.629
LSNS-6 × SOC	0.1	0.037	0.007 **	0.097	0.035	0.006 **

Note. *m* = number of imputations. All continuous variables were standardized (Z-transformed). LSNS-6 = Lubben Social Network Scale–6; SOC = Sense of Coherence; MNA-SF = Mini Nutritional Assessment–Short Form; SE = Standard error. * *p* < 0.05; ** *p* < 0.01; *** *p* < 0.001.

Across additional analytic approaches—including robust regression using Huber’s M-estimator, gamma regression with a log link, and bootstrap resampling—the key predictors remained consistent. The interaction term (LSNS-6 × SOC) remained positive in direction and statistically stable, though marginal in the gamma model (*p* = 0.077). These results collectively support the robustness of the primary findings under diverse model assumptions (Table 4).

**Table 4:** Robustness Checks Using Robust Linear Regression (rlm) and Gamma Regression (glm).

		Robust Linear Model			Gamma Regression Model	
Variable	β	SE	*p*-Value	β	SE	*p*-Value
Intercept	2.999	0.145	<0.001 ***	1.39	0.037	<0.001 ***
LSNS-6	−0.638	0.083	<0.001 ***	−0.142	0.021	<0.001 ***
SOC	−0.261	0.083	0.002 **	−0.051	0.021	0.015 *
MNA-SF	−0.347	0.086	<0.001 ***	−0.09	0.022	<0.001 ***
Age	0.494	0.09	<0.001 ***	0.125	0.023	<0.001 ***
Female (ref: male)	−0.118	0.177	0.503	−0.024	0.045	0.6
Living alone	0.118	0.197	0.549	0.004	0.05	0.929
LSNS-6 × SOC	0.201	0.072	0.006 **	0.033	0.018	† 0.077

Note. All continuous variables were standardized (Z-transformed). For the robust linear regression (rlm) model, *p*-values were computed from *t*-statistics using the *t*-distribution (df ≈ n − k). † *p* < 0.10 (trend-level); * *p* < 0.05; ** *p* < 0.01; *** *p* < 0.001. LSNS-6 = Lubben Social Network Scale–6; SOC = Sense of Coherence; MNA-SF = Mini Nutritional Assessment–Short Form; SE = Standard error.

Model robustness was further confirmed by comparing results across alternative specifications (robust linear and gamma regression). As the pattern of significant and non-significant predictors remained stable across models, inclusion of theoretically relevant covariates (e.g., gender, living arrangement) was maintained to ensure comparability and control for potential confounding.

### 3.5. Bootstrap Validation of the Interaction Effect

Bootstrap analysis based on 1000 replications yielded a 95% confidence interval for the interaction term of [0.086, 0.381]. This result indicates a consistently positive moderating effect of SOC, reinforcing the reliability of the observed interaction.

### 3.6. Visualization of the Interaction Effect

**Figure 1:**
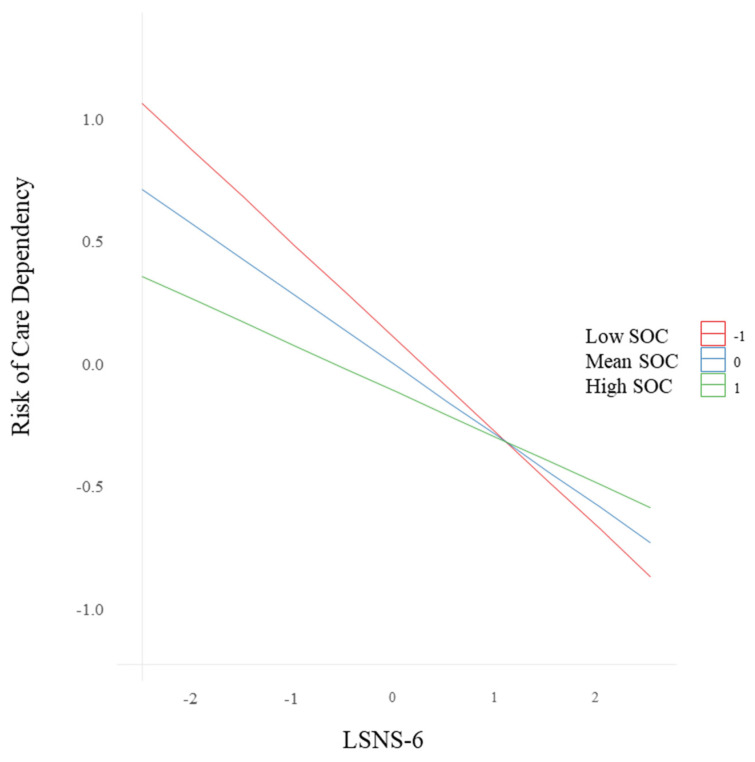
Interaction Between Social Isolation and Sense of Coherence on Care Dependency Risk. Predicted care dependency risk is plotted against LSNS-6 scores at three levels of sense of coherence (SOC): low (Z =−1), average (Z = 0), and high (Z = +1). Estimates were generated from a linear regression model adjusting for MNA-SF, age, sex, and living arrangement, with all covariates fixed at their mean values. A stronger negative association between social isolation and care dependency risk was observed when SOC was low, suggesting increased vulnerability. In contrast, this association was attenuated at higher levels of SOC, supporting its role as a psychological buffer.

Figure 1 illustrates the predicted relationship between LSNS-6 and care dependency risk across SOC levels.

## 4. Discussion

This study examined the association between social isolation and the risk of care dependency among community-dwelling older adults in Japan and explored whether the Sense of Coherence (SOC) moderates this relationship. Consistent with previous research, social isolation was significantly associated with higher care dependency risk [2,3]. Furthermore, SOC moderated this association, suggesting that it functions as a psychological protective resource buffering the adverse effects of social isolation.

### 4.1. Social Isolation and the Risk of Care Dependency

The observed link between social isolation and care dependency risk aligns with existing evidence that insufficient social connection contributes to poor health outcomes through multiple pathways. These include reduced physical activity, unhealthy lifestyle behaviors, and limited instrumental or emotional support. In addition, chronic stress responses associated with social isolation—such as inflammation and altered neuroendocrine activity—may accelerate physiological deterioration and heighten vulnerability. These mechanisms underscore that care dependency risk is multifactorial, involving both physical and social determinants of health.

Interestingly, neither sex nor living alone was significantly related to care dependency risk in this study. One plausible explanation is that participants were relatively healthy and functionally independent. Moreover, household composition may not accurately represent the quality or availability of social support. Previous research indicates that the structure and perceived quality of social networks may exert greater health influence than mere living arrangements [33]. Accordingly, the use of network-based quantitative measures such as the LSNS-6 emphasizes the importance of assessing social connectedness more precisely. These findings suggest that the social context in which older adults live may play a more decisive role in maintaining independence than demographic characteristics such as sex or living arrangement. From a public health perspective, the results underscore the importance of strengthening community-based social networks as a key preventive strategy against care dependency.

### 4.2. The Buffering Role of SOC

A major contribution of this study is the identification of SOC as a moderating factor in the relationship between social isolation and care dependency risk. Rather than directly lowering risk, higher SOC appeared to mitigate the negative effects of isolation, consistent with Antonovsky’s salutogenic model [8], which conceptualizes SOC as a psychological orientation enabling individuals to view stressors as comprehensible, manageable, and meaningful.

In older adulthood, a strong SOC may enhance resilience, facilitating maintenance of health-promoting behaviors even when external social support is limited. However, this buffering effect may depend on the individual’s perception of isolation as a stressor; if isolation is not perceived as threatening, SOC-related coping mechanisms may not be activated.

This finding differs from that of Lin et al. [17], who reported a mediating—but not moderating—role of SOC in the relationship between aging-related self-stereotypes and social isolation. Differences in outcome variables (care dependency vs. perceived isolation), cultural contexts, and analytical design (observational vs. structural modeling) may explain the discrepancy. Although SOC was initially regarded as a stable trait, emerging evidence suggests it can be enhanced through targeted interventions such as psychoeducational programs, reminiscence therapy, and mindfulness training [34]. Such findings highlight SOC as a potentially modifiable psychological resource relevant for preventive strategies. The moderating effect of SOC observed in this study indicates that psychological resilience can partly offset the negative consequences of social isolation. This finding highlights the potential value of incorporating SOC-enhancing interventions—such as stress management programs, group activities fostering meaning, and reflective communication—into community care initiatives for older adults.

### 4.3. Cultural and Practical Implications

Interpretation of these findings should consider Japan’s sociocultural context. Collectivist values emphasizing interpersonal harmony may heighten the psychological distress associated with isolation. Prior research suggests that social isolation exerts stronger psychological effects in interdependent cultures than in individualistic ones [35]. Moreover, older Japanese adults may hesitate to express emotional distress or seek social support, which could amplify isolation’s adverse consequences. These cultural tendencies underline the importance of assessment tools and interventions sensitive to familial and relational dimensions.

From a public-health perspective, integrating SOC-enhancing components into Japan’s community-based integrated care system could strengthen preventive efforts. Combining psychological support and self-reflection-based programs with community-engagement initiatives may simultaneously reduce isolation and reinforce internal coping capacities. Including SOC and social-network assessments such as the LSNS-6 in routine screenings could identify individuals with low resilience and high care dependency risk, enabling early, targeted interventions. The point estimates being close to the lower confidence bounds may indicate sensitivity to model specification and the limited variance of the dependent variable.

### 4.4. Limitations and Future Directions

Several limitations should be acknowledged. First, the cross-sectional design prevents causal inference. Although social isolation may increase the risk of care dependency, the reverse may also be true—individuals with declining function could become more socially isolated. Longitudinal studies are needed to clarify the directionality and causal mechanisms underlying these associations.

Second, reliance on self-reported measures (e.g., SOC, social networks) may have introduced response bias. Third, while the three-item SOC-3-UTHS is validated for older Japanese adults, its brevity may not capture the construct’s full multidimensionality. Fourth, the 15-item Care Prevention Checklist focuses strongly on behavioral and lifestyle aspects but includes fewer items on emotional state compared with the 25-item Basic Checklist, potentially limiting psychological coverage. Fifth, although sex was controlled for in regression models, interaction or stratified analyses were not conducted due to limited sample size; future studies should examine sex-specific differences. Finally, as this research was confined to one neighborhood federation, generalization to other regions or cultures should be made cautiously.

Despite these limitations, this study suggests that social isolation is associated with an increased risk of care dependency and that SOC may play a potential buffering role supporting independent living in later life. Although the explanatory power of the models was modest, the observed trends underscore the importance of psychosocial resources in promoting healthy aging. Incorporating SOC assessment and enhancement into community prevention strategies may contribute to more personalized and sustainable support systems for older adults. To clarify causal mechanisms, future longitudinal and interventional studies are warranted.

## 5. Conclusions

This study suggests that social isolation is significantly associated with an increased risk of care dependency among community-dwelling older adults in Japan. The Sense of Coherence (SOC) may function as a psychological buffer mitigating this negative association, suggesting that enhancing SOC may help maintain independent living and reduce the risk of care dependency. Although the explanatory power of the model was moderate, the observed trends highlight the potential importance of psychosocial resources in supporting independent living and reducing care dependency risk. Integrating SOC assessment and strengthening interventions into community-based preventive care may contribute to a more sustainable and person-centered support system for aging populations.

## Data Availability

The datasets generated and analyzed during the current study are available from the corresponding author upon reasonable request. The data are not publicly available due to ethical and privacy restrictions, as the dataset contains personally identifiable information of older adults and public sharing was not approved by the institutional ethics committee.

## Public Involvement Statement

No public involvement in any aspect of this research.

## Guidelines and Standards Statement

This manuscript was drafted in accordance with the Strengthening the Reporting of Observational Studies in Epidemiology (STROBE) statement for cross-sectional studies.
